# Ureteral Injury by a Retained Knife After Abdominal Trauma: A Case Report

**DOI:** 10.7759/cureus.32719

**Published:** 2022-12-20

**Authors:** Wassim Alaoui Mhammedi, Mohamed Mokhtari, Anouar El Moudane, Ali Barki

**Affiliations:** 1 Urology Department, Mohammed VI University Hospital, Faculty of Medicine and Pharmacy of Oujda, Oujda, MAR

**Keywords:** ureter, genitourinary trauma, foreign body, stab wound, ureteral injury

## Abstract

Isolated ureteral injuries are rare, occurring particularly in gunshot wounds to the abdomen. These are much rarer in the context of stab wounds. These lesions are usually silent. We report a 30-year-old man with a history of abdominal penetrating trauma with a knife, 11 years ago before the actual admission to the urology department. The patient’s report describes a retained metallic foreign body in the right lumbar area. At admission, the patient presented with a four-months history of right lumbago. An abdominal computed tomography scan revealed the presence of a right para-renal small urinoma and identified the 52 x 20 mm metallic foreign body at the level of L3 and L4 vertebral bodies, with the presence of mild right ureterohydronephrosis. Ureteral injuries can lead to significant morbidity and mortality when unrecognized or mismanaged. The basis of therapy for patients with ureteral injuries is to maintain renal drainage with options depending on individual cases.

## Introduction

Ureteral injuries are rare and present 2.5% of all genitourinary traumas. This is due to the ureteral small diameter, and its retroperitoneal location which is protected by the bony pelvis, psoas muscle, and vertebrae [1,2]. Ureteral injuries are usually secondary to iatrogenic trauma during endoscopy, laparoscopy, or open surgery [1]. In terms of non-iatrogenic ureteral injuries, these occur especially (2-5%) following abdominal gunshots [1]. Ureteral lesions during abdominal stab wounds are even rarer [3,4]. Clinically, ureteral injuries are usually silent with no early symptoms [5]. Symptoms are mostly due to associated injuries to other parts of the genito-urinary tract [2]. The early and precise recognition of a ureteral injury is a key diagnostic step since it conditions correct management, otherwise high rates of morbidity and mortality can be observed [2]. Hereby, we report a 30-year-old man with a history of abdominal penetrating trauma 11 years ago. At admission, the patient presented with a four-month history of right lumbago. The abdominal computed tomography scan revealed the presence of right para-renal urinoma and identified the 52 x 20 mm metallic foreign body with the presence of a right ureterohydronephrosis.

## Case presentation

**Figure 1:**
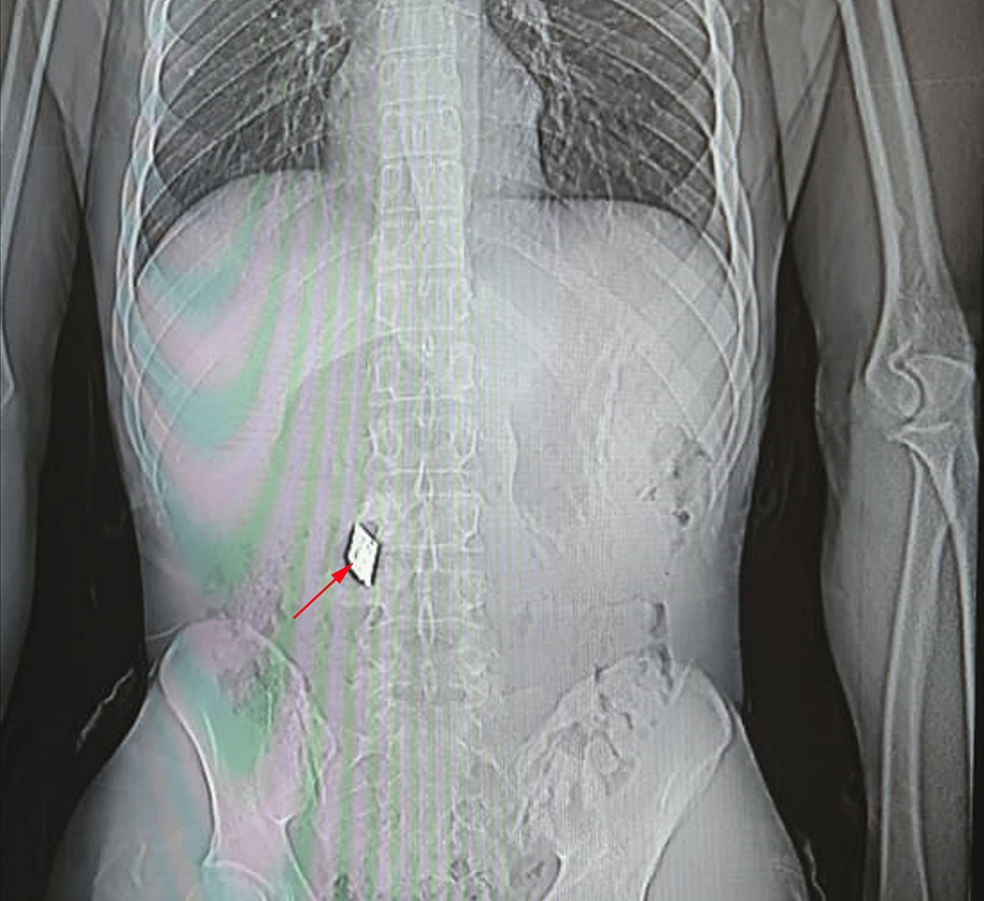
Abdominal radiograph The image reveals the presence of a 50 x 20 mm metallic foreign body (red arrow) at the right lumbar area at the level of L3 and L4 vertebral bodies.

We report a 30-year-old man who had an experience of abdominal penetrating trauma 11 years ago. His report describes a retained metallic foreign body in the right lumbar area at the level of L3 and L4 vertebral bodies. At admission to the urology department, the patient presented with a four-month history of right lumbago, with no pain radiation. The patient reports no fever and no other urological symptoms. Clinical examination revealed stable vital signs: arterial blood pressure at 138/76 mmHg, heart rate at 86 beats per minute, respiratory rate at 15 breaths per minute, and body temperature at 37 degrees Celsius. We noticed an empty right scrotal sac. Laboratory investigations revealed no anemia (hemoglobin at 13.7 g/dl). The white blood count was elevated at 10440/mm^3^, and the platelets count was normal at 413000/ mm^3^. Creatinine blood level was elevated at 18mg/l, and urea level was elevated at 1,64 g/l. Sodium and potassium blood level were slightly low with values at 122 and 2.9 mmol/l, respectively. Rectal examination showed a normal prostate. An abdominal standard radiograph was performed and revealed the presence of an approximately 50 x 20 mm metallic foreign body at the right lumbar area at the level of L3 and L4 vertebral bodies (Figure 1).

**Figure 2:**
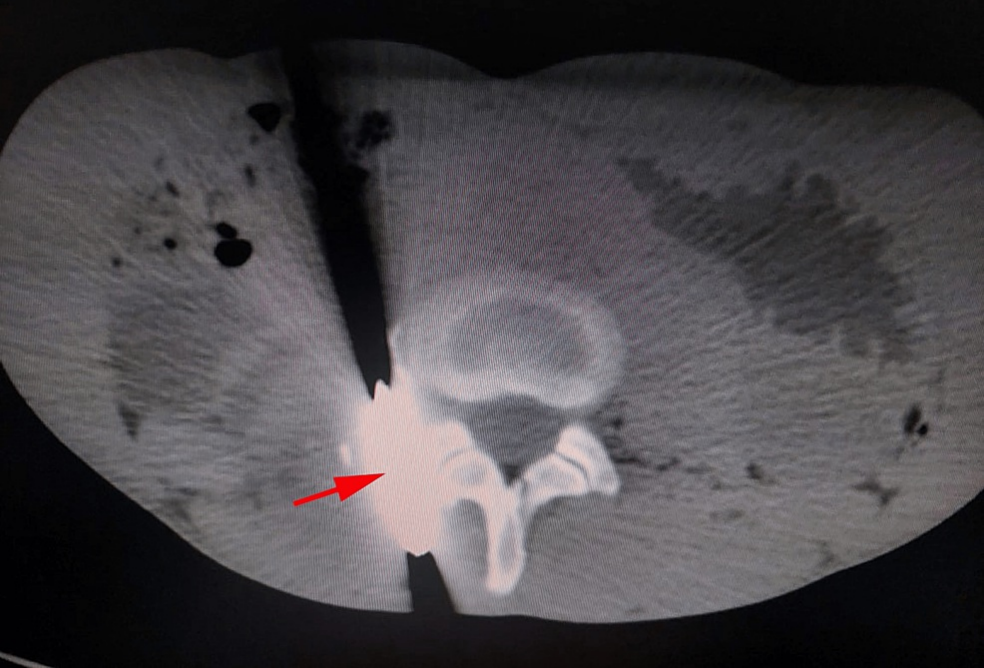
Abdominal computed tomography scan The image reveals the presence of a 52 x 20 mm metallic foreign body in the lumbar region at the level of L3 and L4 vertebral bodies. Exploration of the left kidney and ureteral duct revealed no anomalies.

An abdominal computed tomography scan revealed the presence of right para-renal urinoma and identified the 52 x 20 mm metallic foreign body in the lumbar region at the level of L3 and L4 vertebral bodies, with the presence of a right moderate ureterohydronephrosis. The presence of contrast extravasation identified an injury of the right ureter. Exploration of the left kidney and the ureter revealed no anomalies (Figure 2).

An emergency laparotomy was performed through a right lumbar incision. Intraoperative exploration confirmed the presence of the metallic body, and the presence of a complete section of the right ureter at the level of the L4 vertebral body. The visualized collection on imaging was also identified and compatible with a urinoma secondary to the complete section of the right ureter. A right JJ stent was placed, and a ureteroureterostomy was performed. The urinoma was drained, and extraction of the metallic foreign body was performed. The patients also received amoxicillin and clavulanic acid 250mg every eight hours for 15 days.

Postoperatively, the patient had evolved well. The control biological investigation revealed normal blood creatinine, urea, sodium, and potassium levels. Contrast-enhanced CT scanning was planned every six months and an assessment of renal function (blood creatinine, urea, sodium, and potassium levels) was planned every month. Removal of the JJ probe was planned six months post-operatively.

## Discussion

Ureteral injuries are rare, representing 2.5% of all genitourinary traumas [1,2]. In terms of non-iatrogenic ureteral injuries, these occur especially (2-5%) following abdominal gunshots [1]. Ureteral lesions in the context of stab wounds of the abdomen, as in our reported case, are rare [3,4]. The diagnosis of ureteral lesions, especially when isolated, is classically difficult since there are no early symptoms [1]. Hematuria which can occur as a result of trauma in any segment of the genitourinary tract is not reported in every case of ureteral trauma and is reported in only 43% of cases of isolated ureteral injuries [2,6,7]. Other symptoms of ureteral injury may include hypotension [8]. Due to the paucity of clinical orientation elements, relying on imaging is crucial for an early diagnosis [2].

To our knowledge, no similar cases have been reported in the literature, especially because of the long duration between the initial abdominal stab wound and the revelation of the ureteral injury in form of lumbar pain. We think that the absence of symptoms could be explained by a recent migration of the foreign body towards the ureter.

Retrograde pyelography, which is considered the most accurate imaging technique, enables the identification of the extent and the location of the ureteral injuries. The disadvantages of this technique are essentially related to its cumbersome and time-consuming nature, explaining it when a missed ureteral injury is suspected [9].

In the context of trauma, ultrasound, a technique that has not been used for our patient, has progressively gained great interest. Limitations regarding ultrasounds include difficulty in the detection of different ureteral lesions because of the small caliber and retroperitoneal location of the ureters.

As was performed for our case, contrast-enhanced computer tomography scan remains the most sensitive imaging technique [10,11]. CT scans can detect up to 80% of ureteral injuries. These appear as extravasation of contrast from the ureter as a direct sign or indirectly as unilateral hydronephrosis or as a poor ipsilateral renal excretion [12,13]. CT scan is therefore the gold standard imaging technique in stable patients as recommended by the AUA (American urological association) trauma guidelines [14]. However, in hemodynamically unstable patients, early resuscitation is an obligation. 

The basis of therapy for patients with ureteral injuries is to maintain renal drainage. Options depending on individual cases are related to the extent of the devitalized ureter, the ureteral site of injury, and the presence or absence of other associated lesions. These options include laparotomic surgery: anastomosis, with no tension on the anastomotic margins, and debridement of necrotic parts of the ureter, as well as minimally invasive techniques [12,13]. Percutaneous nephrostomy can also be performed as an urgent, and safe method in cases of severe renal function alteration [12].

## Conclusions

Ureteral injuries are rare because of the small diameter of the ureters and their deep retroperitoneal location. An early diagnosis of these lesions is essential for correct management, otherwise, high morbidity and mortality can be observed. Contrast-enhanced computed tomography is the gold standard imaging technique for the diagnosis of these lesions, indicated in hemodynamically stable patients. The first goal of treatment remains to establish normal renal drainage.
